# Temporal Percolation of the Susceptible Network in an Epidemic Spreading

**DOI:** 10.1371/journal.pone.0044188

**Published:** 2012-09-13

**Authors:** Lucas Daniel Valdez, Pablo Alejandro Macri, Lidia Adriana Braunstein

**Affiliations:** 1 Instituto de Investigaciones Físicas de Mar del Plata—Departamento de Física, Facultad de Ciencias Exactas y Naturales, Universidad Nacional de Mar del Plata, Mar del Plata, Argentina; 2 Center for Polymer Studies, Boston University, Boston, Massachusetts, United States of America; Universidad de Zarazoga, Spain

## Abstract

In this work, we study the evolution of the susceptible individuals during the spread of an epidemic modeled by the susceptible-infected-recovered (SIR) process spreading on the top of complex networks. Using an edge-based compartmental approach and percolation tools, we find that a time-dependent quantity 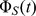, namely, the probability that a given neighbor of a node is susceptible at time 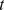, is the control parameter of a node void percolation process involving those nodes on the network not-reached by the disease. We show that there exists a critical time 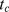 above which the giant susceptible component is destroyed. As a consequence, in order to preserve a macroscopic connected fraction of the network composed by healthy individuals which guarantee its functionality, any mitigation strategy should be implemented before this critical time 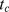. Our theoretical results are confirmed by extensive simulations of the SIR process.

## Introduction

The study of epidemic spreading has been one of the most successful applications on networks science. Recent outbreaks of new influenza strains like the H1N1 [1] and the H5N5 flu or the Severe Acute Respiratory Syndrome (SARS) [2], which are characterized by a high rate of mortality and/or fast propagation velocity, motivate the development of epidemic models that capture the main features of the spread of those diseases. In particular, mathematical tools applied to model epidemics are very important since they allow to understand how a disease impact on the society, helping to develop new policies to slow down its spreading.

One of the simplest models that reproduce seasonal diseases, such as influenza, is the susceptible-infected-recovered (SIR) model [3], [4], which has been the subject of extensive theoretical and numerical research on complex networks [3]. In the SIR model the individuals can be in one of three states, susceptible, infected or recovered. In its discrete formulation [5]–[7], at each time step, infected individuals infect their susceptible neighbors with probability 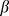 and recover at a fixed time 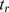 since they were infected, called recovery time. According to these rules, the disease spreads on the contact network until it reaches the steady state where there are only susceptible and recovered individuals. It was found that the steady state of the SIR model can be mapped into a link percolation problem which provides a theoretical framework to study this process [6], [8]–[10]. It is known that the size of the infection, defined as the fraction of recovered individuals at the steady state, is governed by the effective probability of infection or transmissibility 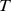 of the disease which depends on 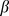 and 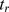. In the SIR model, the size of the infection is the order parameter of a second order phase transition with a critical threshold transmissibility 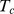. Below 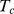 the disease is an outbreak, where the infection reaches a small fraction of the population while above 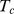 an epidemic develops exactly as in a link percolation process [6], [8]–[10]. In uncorrelated infinite networks this threshold is given by 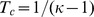 [6], [11], where 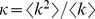 is the branching factor of the network, and 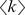 and 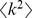 are the first and the second moment, respectively, of the degree distribution 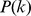. Here, 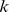 is the degree or number of links that a node can have with 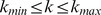. For Erdös-Rényi networks (ER), the degree distribution is 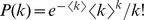 and the threshold is found at 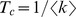. However, most of the real networks have a heterogeneous degree distribution that is better represented by a pure Scale-Free network (SF) with 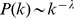, where 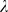 measures the broadness of the distribution. In the thermodynamic limit, for SF networks with 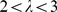, 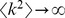 and as a consequence, the critical transmissibility 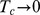 which means that the epidemic spreads for any value of 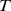 [6], [11]. However, due to finite size effects, real networks have finite critical transmisibilities.

In a recent paper, using a generating function formalism, Newman [12] showed that at the steady state of the SIR model there exists a second threshold 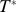 above which the residual network composed by the biggest giant susceptible cluster that remains after a first propagation, is destroyed. From an epidemiological point of view, this implies that if a disease spreads for a second time on the residual network, it cannot become an epidemic. On the other hand, Valdez 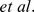 [13] showed that 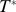 is an important parameter to determine the efficiency of a mitigation or control strategy, because any strategy that decrease the transmissibility below 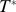, can protect a large and connected cluster of susceptible individuals. Using a percolation framework, they explained the lost of the susceptible giant cluster as a not-random node percolation process, that they called node void percolation, in which a susceptible individual corresponds to a void node in link percolation.

Even though percolation theory was very useful to describe the steady state of the SIR model on complex networks, it is still very challenging to explain the dynamics of the model to develop intervention strategies before the epidemic spreads to a large fraction of the population. To describe the dynamics of epidemic spreading on networks, recently some researchers developed differential rate equations for the SIR model that take into account the network topology. Lindquist 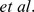 [14] introduced an “effective degree” approach through a large system of ordinary differential equations. Under this approach, the nodes and their neighbors are categorized by their disease state (susceptible, infected, recovered) and each differential equation compute the evolution of the fraction of susceptible or infected nodes with a number 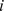 and 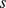 of infected and susceptible neighbors, respectively, with 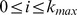 and 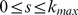. As a result, a system with 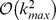 equations needs to be solved. This approach represents accurately the evolution of the number of infected individuals, but at a high computational cost. On the other hand, Miller [15] and Miller 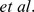 [16], [17] proposed an ingenious approach to describe the evolution of a SIR process with rates by means of an edge-based compartmental model (EBCM) [15], [16] which has the advantage to describe the dynamical spreading of an epidemic with only a few equations. With these equations, the authors found accurate results for the evolution of the number of infected individuals for static and dynamic evolutive topologies like “edge swapping” and “dormant contacts” for transmissibilities above the critical threshold [16].

While most of the literature is focused on studying the evolution of the fraction of infected or susceptible individuals, it has not yet been investigated how the epidemic spread affects the evolution of the network composed by the susceptible individuals. Understanding this problem is important because the network composed by the healthy individuals is the network that sustains the functionality of a society, e.g. the economy of a region. In this paper we present a novel idea for the SIR model, based on a dynamical study of the network composed by susceptible individuals. We show that the temporal decreasing of the size of the giant susceptible cluster can be described as a dynamic void node percolation process with an instantaneous void control parameter. We find that there exists a critical time 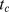 above which the giant susceptible component overcomes a temporal second order phase transition with mean field exponents. The paper is organized as following: in Methods and Results we present the theoretical framework to derive the evolution equations. Then we study the evolution of the giant susceptible cluster and its temporal critical behavior. Finally we present our conclusions.

## Methods and Results

### Theoretical framework

The evolution equations of the dynamic SIR model provide the basis for analyzing theoretically novel magnitudes that could be useful for epidemiologists and authorities to plan policies to stop a disease before an epidemic develops. In the SIR model, initially, all the nodes are susceptible except for one node randomly infected, that represents the index case from which the disease spreads. The infected individual transmits the disease to susceptible neighbors with probability 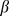 each time unit and recovers 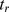 time units since he was infected. For the SIR with fixed recovery time, the transmissibility is given by 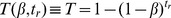 [13].

In order to study the evolution of the states of the individuals in the SIR with fixed recovery time, we use the edge-based compartmental model (EBCM) [15]–[17]. The EBCM is based on a generating function formalism, widely implemented in branching and percolation process on complex networks [3], [18]–[20]. For a branching process that spreads on uncorrelated networks, such as the tree of infected individuals, two generating functions that contain the information of the topology of these networks are defined. The first one is the generating function of the node degree distribution 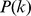 which is given by 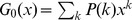. The second one is the generating function of the degree distribution of the first neighbors of a node, also called excess degree distribution 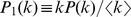, given by 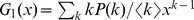. Here, 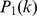 is the probability to reach a neighbor of a node, following a link. It is straightforward that the mean connectivity of the nodes is 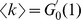.

Denoting the fraction of susceptible, infected and recovered individuals at time 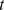 by 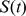, 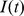 and 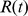, respectively, the EBCM approach describes the evolution of the probability that a node (which we call root node) is susceptible. In order to compute this probability, an edge is randomly chosen and a direction is given, in which the node in the target of the arrow is the root, and the base is its neighbor. Disallowing that the root infects the neighbor, 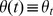 is the probability that the neighbor does not transmit the disease to the root, with 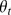 given by
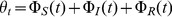
(1)where 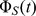, 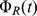 and 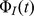 are the probabilities that the neighbor is susceptible, recovered, or infected but has not transmitted yet the disease to the root. The probability that a root node with connectivity 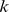 is susceptible is therefore 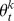 and the fraction of susceptible nodes is 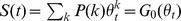. This approach simplifies the calculations, reducing the problem to finding an evolution equation for 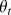, from where the evolution of 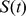, 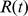 and 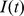 is derived. Thus, using the EBCM approach adapted to SIR with fixed 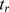 (see Supporting Information Sec.1), the evolutions of 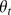, 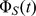 and 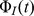 are given by the deterministic equations
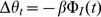
(2)

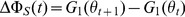
(3)

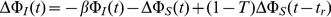
(4)where 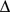 is the discrete change of the variables between times 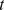 and 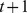. Eq. (2) represents the decrease of 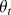 when a infected neighbor transmits the disease. Eq. (3) represents the decrease of 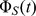 when a susceptible neighbor is infected (notice that 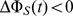). This term contributes to an increase of 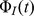 in Eq. (4) where the first term represents the decrease of 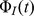 when the links transmit the disease, the second term corresponds to the term of Eq. (3) mentioned above and the third term represents the decrease of 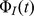 due to the recovery of infected individuals.

From the above equations, the evolution of the fraction of infected individuals can be computed as
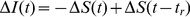
(5)where the first term represents the fraction of new infected individuals (see Supporting Information Sec.1). The second term represents the recovery of infected individuals that have been infected 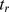 time units ago.

These difference equations correctly describe the evolution of 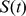, 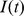 and 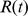 above the criticallity for all values of 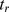 and 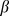 (see Supporting Information Sec.1). In the next section, we will show that combining this approach and dynamic percolation, we can describe the time-dependent evolution of the susceptible individuals in the SIR model as a dynamic void node percolation process for any value of 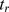.

### Temporal percolation of susceptible individuals

In Ref. [13] it was found that the process under which the susceptible clusters size decrease can be explained with node void percolation defined below that as we will show can be related with the dynamic SIR process.

In the steady state of the SIR model an epidemic cluster is equivalent to a Leath growth process [21], [22] with a link occupancy probability 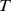. The Leath process on complex networks generates a single cluster that represents the infection tree for a given value of the transmission probability 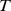. Denoting by 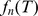 the probability that a cluster reaches the 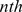 generation following a link, the probability 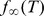 that a link leads to a giant component (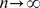) is given by [13], [22]

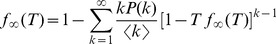
(6)where 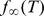 is the solution of
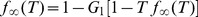
(7)As the “infectious” cluster grows from a root, generation by generation, the sizes of the void clusters, 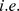 the nodes not reached by the disease, are reduced as in a node dilution process, since when a link is traversed a void cluster loses a node and all its edges. As a consequence, for large generations 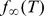 can also be interpreted as the probability that a void cluster loses a node. However, in this kind of percolation process the void nodes are not killed at random, instead they are removed following a link. We call this type of percolation “node void percolation”. If we denote by 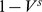 the probability that a void node is removed due to the occupancy of a link, at the steady state the following relation holds
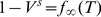
(8)Then 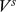 is the probability that a void node is not removed due to the fact that the link has not been traversed. Thus, 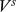 is equivalent to 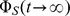 because the void nodes correspond to the susceptible individuals in the steady state. As in any percolation process, there is a critical probability 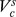 at which the void network undergoes a second order phase transition. Above 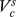 a giant void component exist while at and below 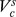 void nodes belong only to finite components. In epidemic terms, this means that at 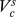 only finite susceptible clusters can be reached. As a consequence, the fraction of links 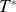 needed to reach this point fulfills [13]

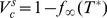
(9)Therefore, from Eqs. (7) and (9) we obtain
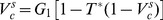
(10)where 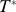 is the solution of Eq. (10). This result shows that at the steady state, for 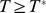, we have 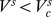 and therefore the size of the giant susceptible cluster 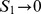 [13]. Even though static percolation is a useful tool to analyze the final size of the giant component of susceptible individuals [12], it is very important to know the evolution of 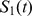, since it can be used as a criteria to begin or to increase an intervention to protect a large fraction of the susceptible population [13]. As we will show below, 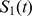 can be fully related with a node void percolation process at every instant 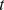.

**Figure 1:**
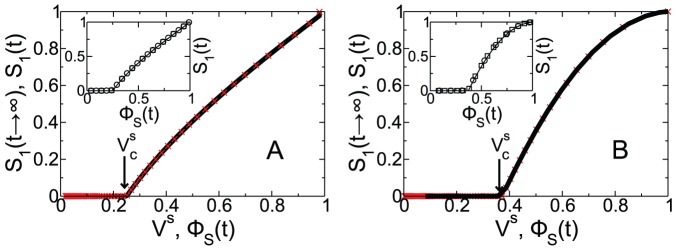
Equivalence between 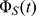 and 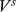. 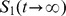 as a function of 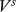 (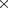) obtained in Refs. [12], [13] and 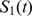 as a function of 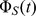 (solid line) obtained from Eqs. (3)–(2) and (12)–(13) with 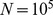 and mean connectivity 4.07 in the giant component for (A) a ER network with 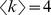 and (B) SF network with 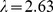, 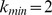 and 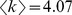. In the insets we show 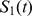 as a function of 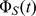 from the simulations (symbols) and from Eqs. (3)–(2) and (12)–(13) (solid line) for 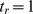 (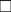) and 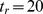 (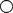). (Color online).

In order to describe the evolution of the size of the giant susceptible cluster, we define 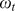 as the probability that a neighbor of a root not connected to the giant susceptible cluster has not yet transmitted the disease to the root at time 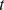. This is possible if the neighbor of the root node is infected but has not yet transmitted the disease, recovered or susceptible but not connected to the giant susceptible cluster, with probabilities 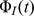, 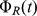 and 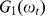 respectively. Similarly to 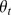 (see Eq. (1)), these probabilities satisfy the relation
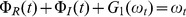
(11)where 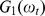 is the generating function of the neighbor of a root not connected to the giant susceptible cluster. From Eq. (1), 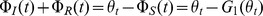. Then Eq. (11) can be rewritten as,
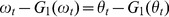
(12)and the evolution of 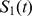 is given by
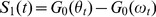
(13)where 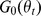 is the total fraction of susceptible individuals and 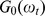 is the fraction of individuals belonging to finite susceptible clusters at time 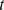. Notice that the dynamical Eqs. (12) and (13) are a time-dependent versions of the ones derived in Ref. [12] for the steady state (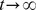) of the SIR model. This suggests that the evolution of the giant susceptible or percolating void cluster can be thought as a temporal percolation process. Thus, the magnitudes derived for the static percolation of the susceptible individuals have a dynamical counterpart. As a result, 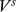 and 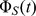, are equivalent not only at the steady state, but also at every instant of time. In order to show the equivalence, in Fig. 1 we show in the same plot 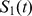 as a function of 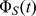, obtained from Eqs. (3)–(2) and (12)–(13), and the steady state 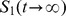 as a function of 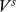 [12] for ER and SF networks with the same 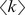 and 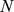 for 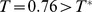.

As we can see, the static curve 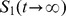 as a function of 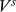 is the same as 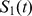 as a function of 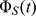 and they coincide with the simulations for different values of 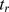 which shows the equivalence between 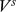 and 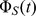 at every instant of time and not only at the steady state (for details of the simulations see Supporting Information Sec.1). Thus our process can be explained by a dynamic percolation with an instantaneous void transmissibility 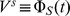.

**Figure 2:**
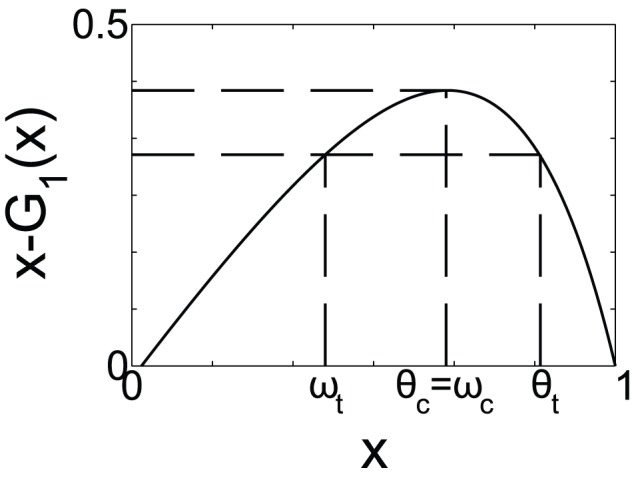
Schematic of the behavior of **Eq. (12)** for 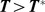
 **.** From the initial condition 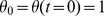, 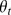 and 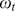, satisfies Eq. (12). For 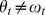 we have two solutions that correspond to 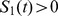. When 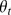 reaches the maximum of the function 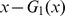, 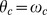, the giant susceptible component is destroyed. The dashed lines are used as a guide to show the possible solutions of Eq. (12).

With our theoretical formulation, we will show that there is a critical time 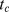 at which the giant susceptible cluster disappears that correspond to the time at which 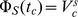. In order to prove this, notice that according to Eq. (12), 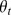 and 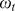 can be thought as two points with the same image of the function 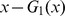. Solving this equation for the variable 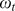 above 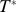, two solutions are found since the curve 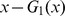 is a concave function for 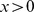 as can be seen in Fig. 2. One of the solutions is the trivial one, for which 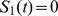, that corresponds to the maximum of the function 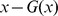 at 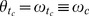. Then the giant susceptible cluster is destroyed at the point 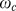 which fulfills
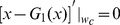
(14)then,
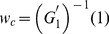
(15)Thus when Eq. (14) is satisfied, the giant susceptible cluster disappears and 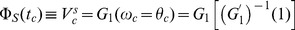, 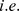


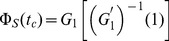
(16)For ER networks it is straightforward to show that 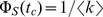.

**Figure 3:**
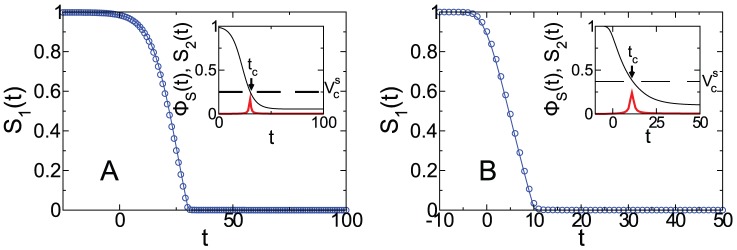
Time evolution of 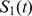 for 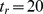 and 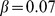 (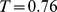) and mean connectivity 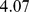 in the giant component for (A) a ER network with 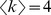 (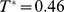) and (B) a SF networks with 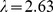, minimal connectivity 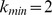 and 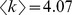 (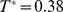). The symbols correspond to the simulations with the time shifted to 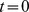 when 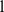
% of the individuals are infected, and the solid lines correspond to the theoretical solutions 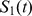 (blue solid line) of Eqs. (12)–(13). In the insets we show the size of the second biggest susceptible cluster 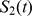 (red solid line) and the evolution of 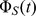 (black solid line) obtained from simulations. The value of 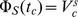 (dashed line) was obtained from Eq. (16). 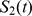 has been amplified by a factor of 50 in order to show it on the same scale as the rest of the curves. The simulations are averaged over 1000 network realizations with 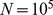. (Color online).

In Fig. 3 we plot the time evolution of the fraction of susceptible individuals 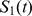 in the susceptible giant component as a function of 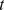 for ER and SF networks obtained from the theory and the simulations, for a transmissibility 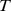 above 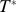.

As shown in Fig. 3, there is an excellent agreement between the theoretical curve 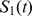, obtained from Eqs. (12) and (13), and the simulations which validate that percolation tools can be used to describe the time dependence of the susceptible individuals in the SIR process for 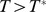. On the other hand, in the figure we can see that for 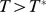, the giant susceptible cluster 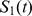 is destroyed at 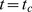 which occurs exactly at 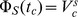 (see the insets of Fig. 3). Our results show that 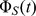 can be used to determine whether a giant susceptible cluster exists at a given time. In turn, in the insets of Fig. 3 we can see that the size of the second susceptible cluster 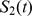 has a sharp peak around 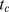, indicating that, as in static percolation, the susceptible individuals overcome a second order phase transition. However, this transition is not given by a random node percolation process. As the disease spreads through the links, the susceptible individuals are removed with probability proportional to 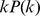, 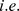, the susceptible network loses the higher degree nodes first. For this reason, the disease spreading induces a second order phase transition in the susceptible network with mean field exponents at 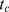 (see discussion in the Supporting Information Sec.2).

An important implication of our results is that, it can be used by the health authorities to implement intervention strategies before the critical time 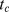 is reached. This will allow to protect a macroscopic fraction of the network composed by healthy interconnected individuals which preserve all the topological properties characteristic of social contact networks and their functionality.

## Conclusions

In this paper we introduce a temporal dynamic percolation to characterize the evolution of the susceptible individuals in a SIR model. We show using an edge-based compartmental approach and percolation tools that as the disease spreads the evolution of the susceptible network can be explained as a temporal node void percolation that can be mapped instantaneously into static percolation. We show that for transmissibilities above 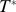, there exist a critical time above which the giant susceptible cluster is destroyed and the susceptible network overcomes a second order transition with mean field exponents. All our theoretical results are in excellent agreement with the simulations. Our findings are very interesting from an epidemiological point of view since the existence of a threshold time implies that when a very virulent disease reaches a small number of susceptible individuals, the authorities have only a limited time to intervene, in order to protect a big community (susceptible giant component) that has not been already reached by the epidemic, and to preserve the topological features of SF networks. Our finding on the susceptible network could be extended to other epidemics dynamics allowing to obtain a better description of the effect of diseases spreading on social and technological networks.

## Supporting Information

Text S1 **(PDF) Supporting Information.**
(PDF)
