# Supplementary material for: Temporal Percolation of the Susceptible Network in an Epidemic Spreading
Source: PLoS One. 2012 Sep 13;7(9):e44188. doi: 10.1371/journal.pone.0044188 (PMC3441612; doi:10.1371/journal.pone.0044188)
Supplement: Text S1 — (PDF) Supporting Information. (PDF) [file pone.0044188.s001.pdf]

Supporting Information

Valdez L. D., Macri P. A., Braunstein L. A.

S1. The EBCM approach

Table S1: Definitions

Variable/Parameter	Definition
β	Infection rate or infection probability.
γ	Recovery rate.
t_r	Recovery time.
T	Transmissibility.
θ_t	Probability that a neighbor of a root node has not transmitted yet the disease to the root node at time t.
$\Phi_S(t)$	Probability that a neighbor of a root node is susceptible at time t.
$\Phi_I(t)$	Probability that an infected neighbor of a root node has not transmitted the disease to the root node at time t.
$\Phi_R(t)$	Probability that a neighbor is recovered at time t without having transmitted the disease to the root node.

In the EBCM approach, θ_t is the probability that a root node has not being infected by a neighbor at time t. This is possible if the neighbor is susceptible, recovered, or infected but has not transmitted the disease yet to the root, which happens with probabilities $\Phi_S(t)$, $\Phi_R(t)$ and $\Phi_I(t)$, respectively. Then, $\theta_t = \Phi_S(t) + \Phi_I(t) + \Phi_R(t)$. The probability that a root node of connectivity k is susceptible is θ^k and the fraction of susceptible nodes is $S(t) = \sum_k P(k)\theta_t^k = G_0(\theta_t)$. On the other hand, a neighbor is susceptible with

probability $\Phi_S(t) = G_1(\theta_t)$. Then in the SIR model with infection and recovery rates [1, 2], the probabilities $\Phi_I(t)$, $\Phi_S(t)$ and θ_t evolve as,

$$\dot{\theta} = -\beta\Phi_I, \tag{S1}$$

$$\dot{\Phi}_S = -\beta G_1'(\theta)\Phi_I, \tag{S2}$$

$$\dot{\Phi}_I = -\beta\Phi_I + \beta G_1'(\theta)\Phi_I - \gamma\Phi_I, \tag{S3}$$

where β and γ are the infection and recovered rates. Eq. (S1) represents the decrease of θ when an infected neighbor transmits the disease. The Eq. (S2) represents the decrease of Φ_S when a susceptible neighbor is infected, which is proportional to $G_1'(\theta)$, *i.e.*, the mean connectivity of the susceptible first neighbors or the excess degree of the susceptible individuals, because when a susceptible individual is infected, all its links except the one used to infected it, can transmit the disease. This term contributes to an increase of Φ_I in Eq. (S3). In Eq. (S3) on the *r.h.s*, the first term represents the decrease of Φ_I when the links transmit the disease, the second term corresponds to the term of Eq. (S2) mentioned above and the third term represents the decrease of Φ_I due to the recovery of infected individuals.

To obtain the evolution of $I(t)$, we use the fact that,

$$\dot{I} + \dot{S} + \dot{R} = 0. \tag{S4}$$

As $\dot{R} = \gamma\, I$ and $\dot{S} = d\left(G_0(\theta)\right)/dt = G_0'(\theta)\dot{\theta} = -\beta\Phi_I G_0'(\theta)$, the evolution of the fraction of infected individuals is given by

$$\dot{I} = \beta G_0'(\theta)\Phi_I - \gamma I, \tag{S5}$$

where the first term represents the decrease of S which is proportional to β, the mean connectivity of susceptible individuals $G_0'(\theta)$ and the probability that an outgoing edge from a root is connected with an infected node that has not transmitted the disease to the root at time t. The second term corresponds to the recovery of infected individuals at a rate γ.

We reformulate the EBCM approach process with discrete time steps, for a fixed

recovery time t_r. It is straightforward that Eq. (S1-S5) can be written as,

$$\begin{aligned} \Delta\theta_t &= -\beta\Phi_I(t), & \text{(S6)} \\ \Delta\Phi_S(t) &= G_1(\theta_{t+1}) - G_1(\theta_t), & \text{(S7)} \\ \Delta\Phi_I(t) &= -\beta\Phi_I(t) - \Delta\Phi_S(t) + (1-T)\Delta\Phi_S(t-t_r), & \text{(S8)} \end{aligned}$$

where $1 - T = (1-\beta)^{t_r}$ denotes the probability that an infected individual has not transmitted the disease to a susceptible individual during t_r time units since he was infected. Finally the evolution of the fraction of infected individuals is given by

$$\Delta I(t) = -\Delta S(t) + \Delta S(t - t_r), \qquad \text{(S9)}$$

where $-\Delta S(t) = -\left(G_0(\theta_{t+1}) - G_0(\theta_t)\right)$ represents the fraction of new infected individuals and the second term represents the recovery of infected individuals that have been infected t_r time units ago.

For the simulations we infect only one individual in the giant component of the network and at each time step all the infected individuals infect their susceptible network with probability β and recover at a fixed time t_r since they were infected. We select only the runs in which the size of the epidemic has reached a macroscopic fraction of individuals in the steady state [3, 4, 5] because the deterministic equations are only valid for epidemics above the critical threshold T_c. We performed all the simulations using synchronized or simultaneous updates at each time step.

In Fig. S1, we plot the time evolution of the fraction of infected nodes $I(t)$ for ER and SF networks obtaining by the EBCM approach Eqs. (S6-S9) and the simulation. For the simulations we shifted $t = 0$ to the instant when the disease has reached 1% of the individuals. We choose this reference time, as the time when the disease has reached a size enough to growth deterministically. This choice compensates the time dispersion of each trial around the theoretical solution due to stochastic effects at the early stages of the process when the number of infected nodes is small [2] (see the insets of Fig. S1). As shown in Figs. S1A-B, each trial simulation has the same shape as the theoretical solution which shows that the EBCM approach and the simulations are in excellent agreement.

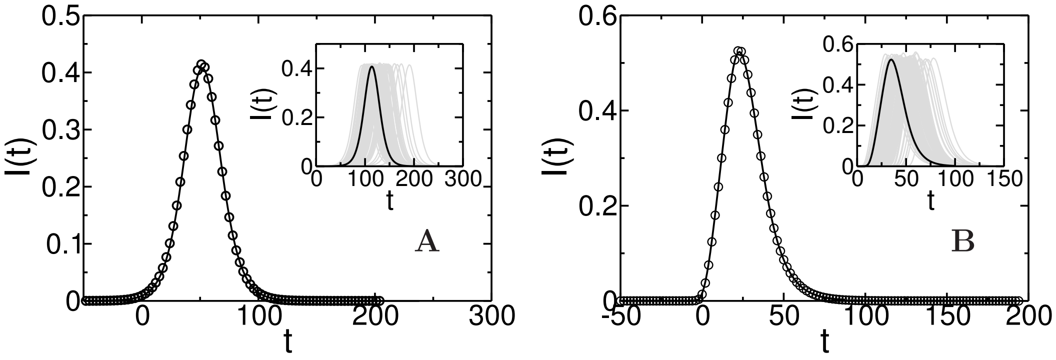

Figure S1: $I(t)$ for epidemics with $t_r = 20$ and $\beta = 0.04$ ($T = 0.55$) on networks with mean connectivity 4.07 in the giant component, for a ER network with $\langle k \rangle = 4$ (A) and a SF with $\lambda = 2.63$, minimal connectivity $k_{min} = 2$ and $\langle k \rangle = 4.07$ (B). The symbols correspond to an average of one hundred different network realizations with $N = 10^5$ nodes and the solid black curve is the numerical solution of Eq. (S9) shifting the curves to $t = 0$ when 1% of the individuals are infected. The insets show the individual 100 network realizations (solid gray lines) and the numerical solution of Eq. (S9) (solid black line) without the temporal shift transformation.

S2. Node void percolation in the time domain

In node void percolation, as a link is traversed, void node is removed. The void nodes are removed with probability proportional to $kP(k)$. As the susceptible nodes can be mapped into void node percolation, the susceptible network loses their higher degree nodes first as in an intentional attack. As a consequence, the resulting susceptible network is more homogeneous than the original. Thus, mean field exponents of a second order percolating phase transition [6] are expected. In order to show the effect of the disease spreading on the highest degree nodes, in Fig. S2 we plot for a SF network the effective degree distribution of the susceptible nodes obtained from the simulations, in which a susceptible node has degree k when it has k susceptible neighbors.

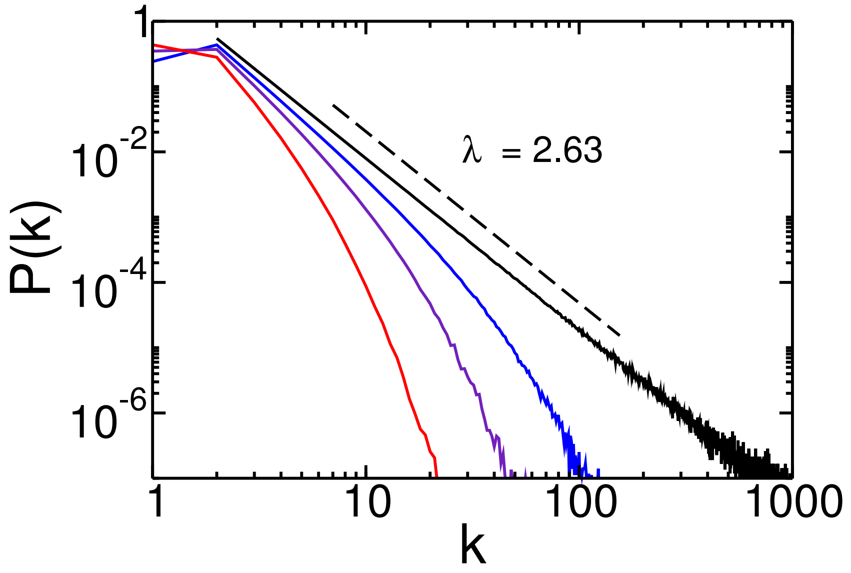

Figure S2: Simulation results of the degree distribution of susceptible nodes for a SF network with $\lambda = 2.63$, $k_{min} = 2$ and $\langle k \rangle = 4.07$ at different times: at the beginning of the spreading (black solid line), when the disease has reached 10% of individuals (blue solid line), 25% of individuals (violet solid line) and 50% of individuals corresponding to t_c (red solid line). (Color online).

As shown in Fig. S2, as the disease spreads, the effective degree distribution loses the heavy tail. As a result of this process, the susceptible clusters becomes more sparse and at the critical time t_c the topology of the susceptible clusters change drastically since the susceptible individuals lose all the hubs and $P(k)$ has an exponential tail. For percolation in mean field it is known that at the criticality the finite cluster size distribution $n_S \sim s^{-\tau}$ with $\tau = 2.5$ and $S_1\left[\Phi_s(t)\right] \sim \Phi_s(t) - \Phi_s(t_c)$. In Fig. S3 we plot the simulations results of the finite size distribution of the susceptible nodes n_s at $t = t_c$.

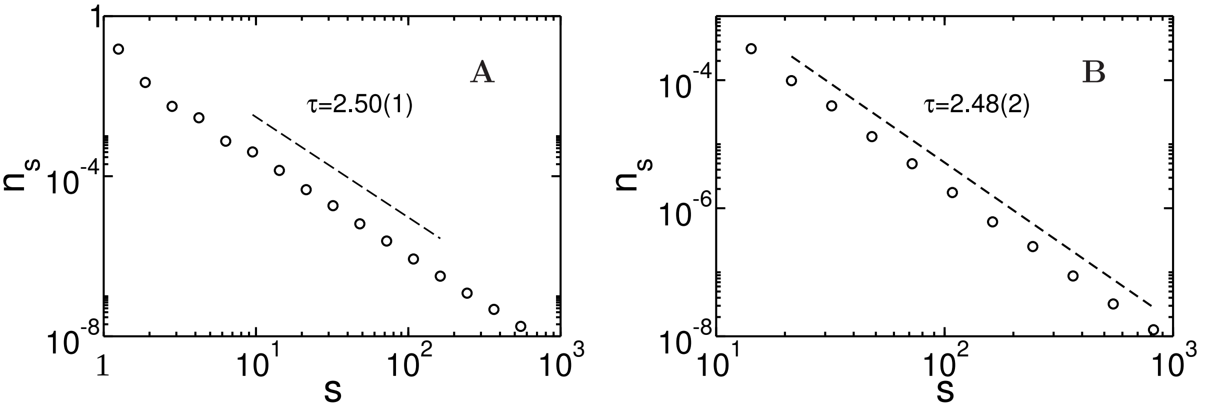

Figure S3: Log-log of the cluster size distribution n_s of finite susceptible clusters (◯) at t_c for $t_r = 20$ and $\beta = 0.07$ (T=0.76) in a ER network with $\langle k \rangle = 4$ for $t_c = 30$(A) and a SF with $\lambda = 2.63$, minimal connectivity $k_{min} = 2$ and $\langle k \rangle = 4.07$ for $t_c = 11$. (B). The dashed line corresponds to a power law fitting, from where we obtain an exponent $\tau \approx 2.5$. Our simulations were averaged over 10000 network realizations with $N = 10^5$.

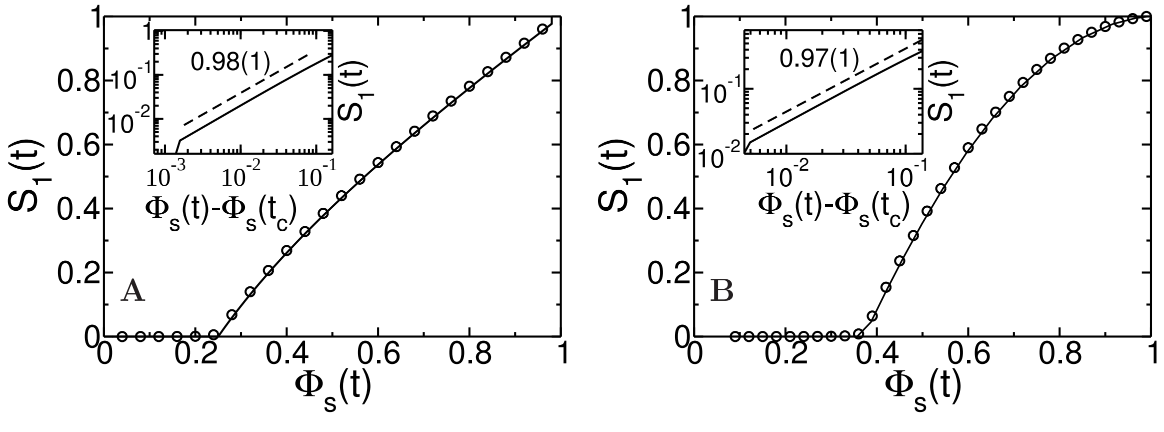

Figure S4: S_1 as a function of $\Phi_s(t)$ obtained from simulations ($\bigcirc$) and from the analytical approach (solid line) in a ER network with $\langle k \rangle = 4$ (A) and a SF with $\lambda = 2.63$, minimal connectivity $k_{min} = 2$ and $\langle k \rangle = 4.07$ (B). In the inset we plot S_1 as a function of the distance of $\Phi_s(t)$ to the criticality $\Phi_s(t_c) = V_c$, in log-log scale. The dashed line corresponds to a power law fitting from where we obtain slope ~ 1. Our simulations were averaged over 1000 network realizations with $N = 10^5$.

We can see that at t_c, $n_s(t_c)$ behaves as a power law with exponent $\tau \approx 2.5$ which corresponds to the mean field value, independently of the initial degree distribution of the network [7]. Similarly, in Fig. S4 we plot $S_1(t)$ as a function of $\Phi_S(t)$ obtained from the simulations and the theoretical approach. We compute $\Phi_S(t)$ from the simulations as the square root of the fraction of edges connecting two susceptible nodes [8]. We can see that $S_1(t)$ behaves as a power law with exponent one with the distance to the criticality $\Phi_s(t_c)$, which also corresponds to the mean field value (see Insets of Fig. S4). Since two critical exponents are sufficient to determine the universality class, the results showed above indicate that in a node void percolation process the susceptible network belongs to the same universality class of mean field percolation and confirms quantitatively the homogenization of the susceptible network during a SIR epidemic spreading.

Finally, in Fig. S5, we plot the critical time t_c, computed from the simulations at $\Phi_S(t) = V_c^s$, as a function of T for different values of t_r. We can see that for the same transmissibility T, when t_r increase, the time to intervene grows since β decrease and

thus the disease spreading is retarded. In turn, when the transmissibility T reaches T^* from above, the critical time t_c grows very fast. This phenomenon is analogous to other second order phase transitions in physics like the relaxation time near the Curie temperature, which are called "critical slowing down" [9, 10], and indicates that that once the transmissibility increases slightly above T^*, the time needed to destroy the giant susceptible cluster decreases very fast.

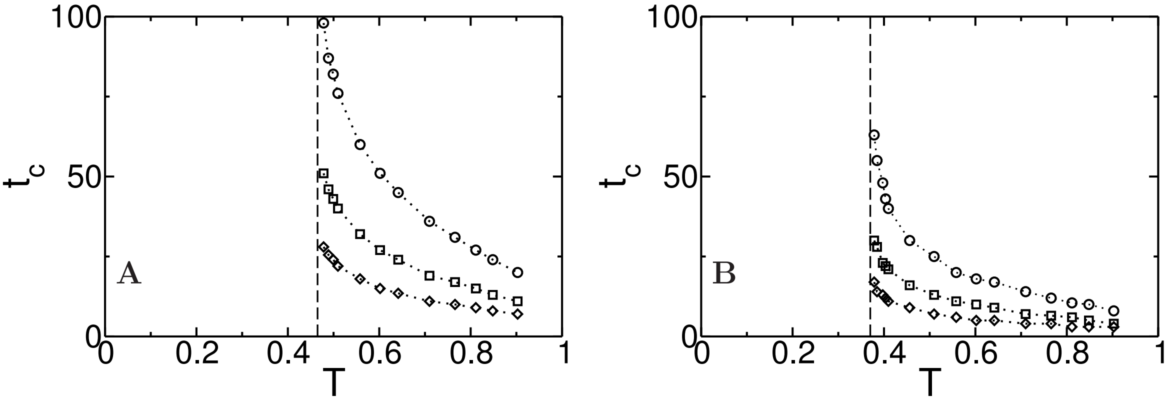

Figure S5: t_c as a function of T for $\beta = 0.07$ and $t_r = 20$ ($\bigcirc$), $t_r = 10$ ($\Box$), $t_r = 5$ ($\diamond$) and mean connectivity 4.07 in the giant component in a ER network with $\langle k \rangle = 4$ ($T^* = 0.46$) (A) and in a SF with $\lambda = 2.63$, minimal connectivity $k_{min} = 2$ and $\langle k \rangle = 4.07$ ($T^* = 0.38$)(B). The dashed line represents the value of T^*. The critical time t_c is measured using $t = 0$ when 1% of individuals are infected. The dotted lines are used as a guide to the eyes.

References

[1] J. C Miller. A note on a paper by Erik Volz: SIR dynamics in random networks. *Journal of Mathematical Biology*, 62:349–358, 2011.

[2] J. C Miller, A. C Slim, and E. M Volz. Edge-based compartmental modelling for infectious disease spread. *Journal of The Royal Society Interface*, 9:890–906, 2011.

[3] C Lagorio, M Migueles, L Braunstein, E López, and P Macri. Effects of epidemic

threshold definition on disease spread statistics. *Physica A: Statistical Mechanics and its Applications*, 388(5):755 – 763, 2009.

[4] M. E. J Newman. Spread of epidemic disease on networks. *Physical Review E*, 66:016128, 2002.

[5] J. C Miller. Epidemic size and probability in populations with heterogeneous infectivity and susceptibility. *Phys. Rev. E*, 76:010101, 2007.

[6] R Cohen, K Erez, D ben-Avraham, and S Havlin. Breakdown of the internet under intentional attack. *Phys. Rev. Lett.*, 86:3682–3685, Apr 2001.

[7] For SF networks with $2 < \lambda < 4$, $\tau = (2\lambda - 3)/(\lambda - 2)$ and the exponent of the order parameter is $1/(3 - \lambda)$ for $2 < \lambda < 3$, $1/(\lambda - 3)$ for $3 < \lambda < 4$ and one for $\lambda > 4$.

[8] As $\Phi_S(t)$ is the probability that a randomly chosen stub belongs to a susceptible node (conditional on the assumption that stub has not transmitted infection to the node) then the probability that both stubs in a random edge belong to susceptible nodes is $(\Phi_S(t))^2$.

[9] D Stauffer and A Aharony. *Introduction to percolation theory.* Taylor & Francis, 1985.

[10] A Bunde and S Havlin. *Fractals and Disordered Systems.* Springer, 1996.
